# Correction: Rossignol, D.A.; Frye, R.E. Cerebral Folate Deficiency, Folate Receptor Alpha Autoantibodies and Leucovorin (Folinic Acid) Treatment in Autism Spectrum Disorders: A Systematic Review and Meta-Analysis. *J. Pers. Med.* 2021, *11*, 1141

**DOI:** 10.3390/jpm12050721

**Published:** 2022-04-29

**Authors:** Daniel A. Rossignol, Richard E. Frye

**Affiliations:** 1 Rossignol Medical Center, 24541 Pacific Park Drive, Suite 210, Aliso Viejo, CA 92656, USA; 2 Barrow Neurological Institute at Phoenix Children’s Hospital, Phoenix, AZ 85016, USA; rfrye@phoenixchildrens.com; 3 Department of Child Health, University of Arizona College of Medicine, Phoenix, AZ 85004, USA

## Error in Table

In the original publication [1], there was a mistake in **“Table 4”** as published. **“The header for Frye et al., 2018 was missing in the original table and the shading was incorrect in the header”.** The corrected **“Table 4”** appears below. The authors apologize for any inconvenience caused and would like to clarify that the scientific conclusions are unaffected. This correction has been approved by the Academic Editor. The original publication has also been updated.

**Table 4:** Outcome measures represented in effect size in key studies which have used *d,l*-leucovorin. Cohen’s d’ was calculated for studies which provided enough information to make such calculations. For controlled studies, the effect size represented the difference between the treatment and the control group, whereas for uncontrolled studies, the effect size was calculated only for the treatment. Effect sizes were considered small if Cohen’s d’ was 0.2; medium for Cohen’s d’ of 0.5, and large if Cohen’s d’ was 0.8. Effects in bold are statistically significant.

Core ASD Symptoms	Communication	Behaviors	Other Symptoms
***d,l*-leucovorin Only Studies**			
**Frye et al., 2013 [37] (Non-Treated Wait List Controlled)**			
**Stereotypy d’ = 1.02**	**Verbal Comm d’ = 0.91** **Expressive Lang d’ = 0.81** **Receptive Lang d’ = 0.76**	**Attention d’ = 1.01** Hyperactivity d’ = 0.25	
**Frye et al., 2018 [72] (Double Blind Placebo Controlled)**			
**ABC Social Withdrawal d’ = 0.27** **ABC Stereotypy d’ = 0.60**	**Verbal Comm (All) d’ = 0.70** **Verbal Comm (FRAA+) d’ = 0.91**	**Hyperactivity d’ = 0.05**	
**Renard et al., 2020** [74] **(Single Blind Placebo Controlled)**			
**ADOS total score d’ = 1.16** **Social interaction d’ = 1.11** **SRS Total d’ = 0.03**	**Communication d’ = 0.66**		
***d,l*-leucovorin Combined with Other Supplements**			
**Adams et al., 2011 [73] (** **Double Blind Placebo Controlled** **; PGI-R Outcome)**			
Play d’ = 0.23 Sociability d’ = 0.15	Expressive Language d’ = 0.37 **Receptive Language d’ = 0.44**	**Hyperactivity d’ = 0.60** **Tantrums d’ = 0.53**	Cognition d’ = 0.34 Gastrointestinal d’ = 0.30 Sleep d’ = 0.18
**Adams et al., 2018** [76] **(Prospective Non-Treatment Controlled; PGI-2 Outcome)**			
**Play d’ = 1.50** **Sociability d’ = 1.44** **Eye Contact d’ = 1.41** **Perseveration d’ = 1.33** **Sound Sensitivity d’ = 1.14**	**Expressive Language d’ = 1.60** **Receptive Language d’ = 1.99**	**Attention d’ = 1.19** **Hyperactivity d’ = 1.46** **Tantrums d’ = 1.00** **Aggression d’ = 0.96** Self-Injury d’ = 0.69	**Cognition d’ = 1.49** **Gastrointestinal d’ = 2.09** **Sleep d’ = 0.92** **Mood d’ = 1.58** **Anxiety d’ = 0.95**
**Ramaekers et al., 2019** [44] **(Baseline Controlled)**			
**CARS d’ = 1.01–1.32**			
**Frye et al., 2013** [79] **(Baseline Controlled)**			
**Interpersonal d’ = 0.43** **Play d’ = 0.59** **Coping d’ = 0.66**	**Expressive Language d’ = 0.59** **Receptive Language d’ = 0.97** **Written Language d’ = 0.56**		**Personal d’ = 0.65** **Domestic d’ = 0.37** **Community d’ = 0.52**
