# Supplementary material for: Cerebral Folate Deficiency, Folate Receptor Alpha Autoantibodies and Leucovorin (Folinic Acid) Treatment in Autism Spectrum Disorders: A Systematic Review and Meta-Analysis
Source: J Pers Med. 2021 Nov 3;11(11):1141. doi: 10.3390/jpm11111141 (PMC8622150; doi:10.3390/jpm11111141)
Supplement: Supplementary file 1 [file jpm-11-01141-s001.zip › jpm-1435696-supplementary.pdf]

Table S1. PRISMA Checklist

Section and Topic	Item #	Checklist item	Location where item is reported
**TITLE**			
Title	1	Identify the report as a systematic review.	Page 1
**ABSTRACT**			
Abstract	2	See the PRISMA 2020 for Abstracts checklist.	Page 1
**INTRODUCTION**			
Rationale	3	Describe the rationale for the review in the context of existing knowledge.	Page 1-3
Objectives	4	Provide an explicit statement of the objective(s) or question(s) the review addresses.	Page 3
**METHODS**			
Eligibility criteria	5	Specify the inclusion and exclusion criteria for the review and how studies were grouped for the syntheses.	Sec 2.2
Information sources	6	Specify all databases, registers, websites, organisations, reference lists and other sources searched or consulted to identify studies. Specify the date when each source was last searched or consulted.	Sec 2.1
Search strategy	7	Present the full search strategies for all databases, registers and websites, including any filters and limits used.	Sec 2.1
Selection process	8	Specify the methods used to decide whether a study met the inclusion criteria of the review, including how many reviewers screened each record and each report retrieved, whether they worked independently, and if applicable, details of automation tools used in the process.	Sec 2.2
Data collection process	9	Specify the methods used to collect data from reports, including how many reviewers collected data from each report, whether they worked independently, any processes for obtaining or confirming data from study investigators, and if applicable, details of automation tools used in the process.	Sec 2.2
Data items	10a	List and define all outcomes for which data were sought. Specify whether all results that were compatible with each outcome domain in each study were sought (e.g. for all measures, time points, analyses), and if not, the methods used to decide which results to collect.	Sec 2.2
	10b	List and define all other variables for which data were sought (e.g. participant and intervention characteristics, funding sources). Describe any assumptions made about any missing or unclear information.	Sec 2.2
Study risk of bias assessment	11	Specify the methods used to assess risk of bias in the included studies, including details of the tool(s) used, how many reviewers assessed each study and whether they worked independently, and if applicable, details of automation tools used in the process.	Sec 2.2
Effect measures	12	Specify for each outcome the effect measure(s) (e.g. risk ratio, mean difference) used in the synthesis or presentation of results.	Sec 2.3
Synthesis methods	13a	Describe the processes used to decide which studies were eligible for each synthesis (e.g. tabulating the study intervention characteristics and comparing against the planned groups for each synthesis	Sec 2.2, 2.3

Section and Topic	Item #	Checklist item	Location where item is reported
		(item #5)).	
	13b	Describe any methods required to prepare the data for presentation or synthesis, such as handling of missing summary statistics, or data conversions.	Sec 2.3
	13c	Describe any methods used to tabulate or visually display results of individual studies and syntheses.	Sec 2.3
	13d	Describe any methods used to synthesize results and provide a rationale for the choice(s). If meta-analysis was performed, describe the model(s), method(s) to identify the presence and extent of statistical heterogeneity, and software package(s) used.	Sec 2.3
	13e	Describe any methods used to explore possible causes of heterogeneity among study results (e.g. subgroup analysis, meta-regression).	Sec 2.3
	13f	Describe any sensitivity analyses conducted to assess robustness of the synthesized results.	Sec 2.3
Reporting bias assessment	14	Describe any methods used to assess risk of bias due to missing results in a synthesis (arising from reporting biases).	Sec 2.3
Certainty assessment	15	Describe any methods used to assess certainty (or confidence) in the body of evidence for an outcome.	Sec 2.3
**RESULTS**			
Study selection	16a	Describe the results of the search and selection process, from the number of records identified in the search to the number of studies included in the review, ideally using a flow diagram.	Sec 2.2, Fig 1
	16b	Cite studies that might appear to meet the inclusion criteria, but which were excluded, and explain why they were excluded.	Sec 2.2
Study characteristics	17	Cite each included study and present its characteristics.	Supplementary material
Risk of bias in studies	18	Present assessments of risk of bias for each included study.	Sec 2.2
Results of individual studies	19	For all outcomes, present, for each study: (a) summary statistics for each group (where appropriate) and (b) an effect estimate and its precision (e.g. confidence/credible interval), ideally using structured tables or plots.	Tables 1-3
Results of syntheses	20a	For each synthesis, briefly summarise the characteristics and risk of bias among contributing studies.	Sec 3.1<br>Sec 3.2.
	20b	Present results of all statistical syntheses conducted. If meta-analysis was done, present for each the summary estimate and its precision (e.g. confidence/credible interval) and measures of statistical heterogeneity. If comparing groups, describe the direction of the effect.	Tables 1-3
	20c	Present results of all investigations of possible causes of heterogeneity among study results.	Tables 1-3
	20d	Present results of all sensitivity analyses conducted to assess the robustness of the synthesized results.	Tables 1-3
Reporting biases	21	Present assessments of risk of bias due to missing results (arising from reporting biases) for each	N/A

Section and Topic	Item #	Checklist item	Location where item is reported
		synthesis assessed.	
Certainty of evidence	22	Present assessments of certainty (or confidence) in the body of evidence for each outcome assessed.	Sec 3.1.<br>Sec 3.2.
**DISCUSSION**			
Discussion	23a	Provide a general interpretation of the results in the context of other evidence.	Page 15
	23b	Discuss any limitations of the evidence included in the review.	Page 19-20
	23c	Discuss any limitations of the review processes used.	Page 20
	23d	Discuss implications of the results for practice, policy, and future research.	Sec 5
**OTHER INFORMATION**			
Registration and protocol	24a	Provide registration information for the review, including register name and registration number, or state that the review was not registered.	Not Registered
	24b	Indicate where the review protocol can be accessed, or state that a protocol was not prepared.	Not Registered
	24c	Describe and explain any amendments to information provided at registration or in the protocol.	Not Registered
Support	25	Describe sources of financial or non-financial support for the review, and the role of the funders or sponsors in the review.	Page 20
Competing interests	26	Declare any competing interests of review authors.	Page 20
Availability of data, code and other materials	27	Report which of the following are publicly available and where they can be found: template data collection forms; data extracted from included studies; data used for all analyses; analytic code; any other materials used in the review.	Page 3-4

Table S2. Studies on Children with CFD where ASD was found to be a characteristic

Study, year	Children	Study and dose	Outcomes	Etiology	Comment
Ramaekers et al., 2004 (Ramaekers and Blau 2004)	20 children with CFD (7 with ASD), ages 2-17 yo	Prospective, open label, *d,l*-leucovorin 0.5-1 mg/kg/day for at least one year	Treatment normalized 5MTHF CSF levels in 18 patients (90%); In children under 6 yo, “marked neurological recovery and cessation of seizures” was observed	No mutations in exons of genes for FRa, FRb; FRα autoantibodies not measured; Mitochondrial not tested	Transient tics and agitation in 2 children (10%); no other adverse events
Ramaekers et al., 2005	28 patients with CFD, ages 2-19	Prospective, open label, *d,l*-	One patient with ASD “recovered completely.” Two	FRα Positive in 4 of 5 children with ASD (80%);	

(Ramaekers, Rothenberg et al. 2005)	yo; 5 patients with ASD	leucovorin 0.5-1 mg/kg/day	other children with ASD had improvements in communication skills and neurologic abnormalities.	Folate Gene Not Sequenced; Mitochondrial not tested	
Moretti et al., 2008 (Moretti, Peters et al. 2008)	7 children with CFD; 5 that could be evaluated for ASD were positive; assessment no possible in other 2	*d,l*-leucovorin 0.5-1 mg/kg/day	Increases in CSF 5MTHF levels; cognitive, motor, social and neurological improvements; reductions in seizures; 3 patients with no clinical improvements	FRα Ab not tested<br>Mitochondrial not tested<br>No mutations in exons of genes for Fra, FRb, PCFT, MTHFR, DHFR, FTCD	One child in this study previously reported (Moretti, Sahoo et al. 2005)
Ramaekers et al., 2008 (Ramaekers, Sequeira et al. 2008)	24 children with CFD, 10 had ASD	Prospective, open label, *d,l*-leucovorin 0.5-2.5 mg/kg/day	5MTHF in CSF normalized; clinical improvements	Blocking FRα Positive in 10 of 10 with ASD (100%)	Additional improved beyond *d,l*-leucovorin by using a milk free diet
Al-Baradie et al., 2014 (Al-Baradie and Chaudhary 2014)	2 children with CFD, 1 with ASD	*d,l*-leucovorin, 0.75-1.7 mg/kg/day	Child became seizure-free and improvements in social interaction and gait; breakthrough seizure when he ran out of *d,l*-leucovorin for 2 weeks	Homozygous novel mutation c.398C>A (p.Pro133His) in the FOLR1 gene;<br>FRα Ab not tested<br>Mitochondrial not tested	Other child without ASD had "dramatic EEG and neurological improvement like her brother."

Table S3. Studies on Children with ASD where CSF was measured for possible CFD

Study, year	Children	Study and dose	Outcomes	Etiology
Ramaekers et al., 2007 (Ramaekers, Blau et al. 2007)	25 children with early-onset and low functioning ASD, ages 2-12 yo; 23 with CFD	Prospective, open label, *d,l*-leucovorin 1-3 mg/kg/day	5MTHF in CSF normalized; 2 children "cured with full recovery from ASD and neurological deficits." Improvements in social interaction (31%), communication (69%) and stereotypy (46%)	Blocking FRα Positive in 19 of 23 children with CFD (83%); no mutations in exons of Fra and FRb.
Moretti et al., 2005 (Moretti, Sahoo et al. 2005)	6 yo girl with ASD	*d,l*-leucovorin, 0.5-1 mg/kg/day	CSF 5MTHF normalized; improvements in motor skills and neurological findings	No mutations in RFC, FBP1; ETC function unremarkable; FRα not tested

Frye et al., 2011 (Frye and Naviaux 2011)	5 children with regressive autistic disorder and Complex IV Overactivity, 2 with CSF testing (without folate supplementation) had CFD	*d,l*-leucovorin, 2 mg/kg/day	Improvements in expressive and receptive language and attention in one child with FRα autoantibodies	Complex IV Overactivity<br>Folate Genes Not Sequenced<br>Fab positive in one and negative in other
Shoffner et al 2010 (Shoffner, Hyams et al. 2010).	28 children with ASD and mitochondrial disease, 22 with CSF measurements	Not Treated		All with mitochondrial disease.<br>Folate Genes Sequencing not reported<br>FRAT Measurement not reported
Frye et al., 2013 (Frye, Sequeira et al. 2013)	98 children with autism spectrum disorder, 16 with CSF measurements	*d,l*-leucovorin, 2 mg/kg/day	0 of 16 (0%) had CFD but all CSF 5-MTHF values were below average.	Folate Genes Not Sequenced<br>All patient that underwent CSF measurements were Fab positive.
Shoffner et al 2016 (Shoffner, Trommer et al. 2016)	67 children with autistic disorder, 2y-11y, overall 11 with CFD.	Not Treated	5 of 67 (7%) had CFD on first examination and 1-3 years later 7 of 31 (23%) had CFD. Overall, 11 of 67 had CFD at some time point (16%)	Mitochondrial Activity Not Measured; Folate Genes Not Sequenced; FRAT Measured using non-validated assay
Ramaekers et al., 2020 (Ramaekers,	38 children with autism (mean age 7.25±3.9 yo)	Not Treated	8/38 (21%) had CFD	FRAT positive in 26/38 (68%)

Sequeira et al. 2020)				
Kanmaz et al 2021 (Kanmaz, Simsek et al. 2021)	1 Child with CFD and ASD	*d,l*-leucovorin, 0.5-9.0 mg/kg/day orally, then *d,l*-leucovorin, 6mg/kg Q6 x 1d IV monthly for 6 months then *d,l*-leucovorin, 6mg/kg IV weekly for 6 months	CSF folate level normalized and severity and frequency of seizures decreased. Eye contact, gross motor and cognitive skills significantly improved; Valproic acid, clonazepam and rufinamide added	Homozygous missense (c.655A>G) in the FOLR1 gene;<br>FRα Ab not tested<br>Mitochondrial not tested

Table S4. Studies of FRα autoantibodies in ASD, by year published

Study	ASD Child % Blocking, Average (SD) Titer	ASD Child %Binding Average (SD) Titer	ASD Child % Either	Parent % Blocking Average (SD) Titer	Parent %Binding Average (SD) Titer	Parent %Either Average (SD) Titer	Sibling %Blocking Average (SD) Titer	Sibling %Binding Average (SD) Titer	Sibling %Either	Control % Blocking Average (SD) Titer	Control %Binding Average (SD) Titer	Control % Either
Frye et al., 2013 (Frye, Sequeira et al. 2013)<br>Frye et al., 2014 (Frye, Sequeira et al. 2014)	56/93 (60%) 0.36 (0.40)	41/93 (44%) 0.55 (0.73)	70/93 (75%)	10/27 (37%) 0.11 (0.15)	2/27 (7%) 0.13 (0.23)	12/27 (44%)	1/6 (17%) 0.07 (0.143)	1/6 (17%) 0.15 (0.26)	2/6 (33%)			
Ramaekers et al., 2013 (Ramaekers, Quadros et al. 2013)	35/75 (47%) 0.21 (0.27)			Mom 19/74 (26%) Dad 9/50 (18%)						Child *1/30 3% Mom 1/30 (3%)		

										Dad<br>1/30<br>(3%)		
Frye et al., 2016 (Frye, Delhey et al. 2016)<br>Frye et al., 2017 (Frye, Wynne et al. 2017)<br>Frye et al 2018 (Frye, Slattery et al. 2018).	16/94<br>(17%)<br>0.09<br>(0.24)	48/94<br>(51%)<br>(0.44)<br>0.63	54/94<br>(58%)									
Quadros et al., 2018 (Quadros, Sequeira et al. 2018)	33/81<br>(41%)<br>3.01<br>(4.95)	44/82<br>(54%)<br>0.27<br>(0.32)	62/82<br>(76%)	Mom<br>21/70<br>(31%)<br>Dad<br>29/65<br>(45%)<br>0.11<br>(0.15)	Mom<br>25/70<br>(36%)<br>Dad<br>29/65<br>(45%)<br>0.13<br>(0.23)	Mom<br>41/70<br>(59%)<br>Dad 45/65<br>(69%)	23/53<br>(44%)<br>2.78<br>(4.89)	29/53<br>(55%)<br>0.28<br>(0.35)	40/53<br>(75%)	2/52<br>(4%)<br>0.08<br>(0.04)	14/52<br>(27%)<br>0.11<br>(0.21)	15/52<br>(29%)
Ramaekers et al., 2019 (Ramaekers, Sequeira et al. 2019)			(1)<br>60/84<br>(71%)<br>(2)<br>62/82<br>(76%)			Cohort 1<br>30.8%<br>Maternal,<br>27.4%<br>Paternal<br>Cohort 2<br>**Mom<br>23/66<br>(35%)<br>Dad 18/66<br>(27%)						DD<br>1/30<br>(3%)
Ramaekers et al., 2020 (Ramaekers, Sequeira et al. 2020)	26/38<br>(68%)	6/17<br>(35%)				12/29<br>(41%)						1/30<br>(3%)
**Total**	140/343<br>(41%)	133/269<br>(49%)	248/351<br>(71%)	All<br>88/286<br>(31%)<br>Mom<br>40/144<br>(28%)	All 56/162<br>(35%)<br>Mom<br>25/70<br>(36%)	All<br>139/294<br>(47%)<br>Mom<br>64/136<br>(47%)	24/59<br>(41%)	30/59<br>(51%)	42/59<br>(71%)	DD Child<br>1/30<br>(3%)<br>DD Mom<br>1/30<br>(3%)	TD 14/52<br>(27%)	TD<br>15/52<br>(29%)<br>DD<br>1/30<br>(3%)

				Dad 38/115 (33%)	Dad 29/65 (45%)	Dad 63/131 (48%)				DD Dad 1/30 (3%) TD Child 2/52 (4%)		

* Controls for this study had developmental delays; **values derived from Table 2 of publication

Table S5. Response to d,l-leucovorin in children with CFD with and without ASD (Total cases with symptoms / total Responses)

	Ramaekers et al 2008 (Ramaekers, Sequeira et al. 2008)		Ramaekers et al 2007 (Ramaekers, Blau et al. 2007)	Moretti et al 2008 (Moretti, Peters et al. 2008)		Ramaekers et al 2005 (Ramaekers, Rothenberg et al. 2005)		Al-Baradie et al 2014 (Al-Baradie and Chaudhary 2014)		Kanmaz et al 2021(Kanmaz, Simsek et al. 2021)
	ASD (n=10)	No ASD (n=14)	All ASD (n=25)	ASD (n=5)	No ASD (n=2)	ASD (n=5)	No ASD (n=23)	ASD (n=1)	No ASD (n=1)	ASD (n=1)
Dose	0.5-2.5 mg/kg/day	0.5-2.5 mg/kg/day	1-3 mg/kg/day	0.5-1 mg/kg/day	0.5-1 mg/kg/day	0.5-1 mg/kg/day	0.5-1 mg/kg/day	0.75-1.7 mg/kg/day	2 mg/kg/day	6mg/kg IV weekly
Irritability/Unrest/Sleep	10/4	9/2	17/12		1 / 0	5 / 3	16 / 14			
Autism	10/6		19/16	5 / 1		5 / 2		1 / 1		1 / 1
Ataxia/Motor	10/7	14/7	17/16	3 / 3		4/4	13/12	1/1		
Pyramidal	3/0	7/0	8/8			4 / 4	12 / 9	1 / 1		
Dyskinesias/Movement	2/1	2/1	7/3	5 / 1	2/0	2 / 2	4 / 0			
Epilepsy	3/1	6/1	10/9	4 / 2	2 / 0	1 / 1	5 / 5	1 / 1	1 / 1	1 / 1

Table S6. Studies examining only *d,l*-leucovorin in ASD, by year published

Study, year	Children	Study and dose	Outcomes	Comment
Frye et., 2013 (Frye, Sequeira et al. 2013)	44 children with ASD, ages 2-15 yo; 9 children with ASD in a wait-list control group	Prospective, case-control study, *d,l*-leucovorin 2 mg/kg/day (max 50 mg per day) over a mean period of 4 months	Improvements in verbal communication (p=0.011), expressive language (p=0.026), receptive language (p=0.017),	Improvement compared to a wait-list control group; Minimal adverse effects noted

			attention (p=0.05) and stereotypy (p=0.009)	
Frye, 2018 (Frye, Slattery et al. 2018)	48 children with ASD (mean age 7 yo; 82% male)	Double-blind, placebo-controlled; 12 weeks of *d,l*-leucovorin 2 mg/kg/day (max 50 mg/day) in 23 children; placebo in 25 children	Improvement in verbal communication (p=0.02) with medium-large effect size; VABS daily living (p=0.05); ABC irritability (p=0.04), ABC social withdrawal (p=0.02), ABC stereotypy (p=0.007), ABC hyperactivity (p=0.02), and ABC inappropriate speech (p=0.004); ASQ stereotypy (p=0.02)	Children with FRα autoantibodies had more significant improvements; no significant differences in adverse events in 2 groups
Renard, 2020 (Renard, Leheup et al. 2020)	19 children with ASD	Single-blind, placebo-controlled; *d,l*-leucovorin 0.29-0.63 mg/kg/day in 9 children; placebo in 10 children	ADOS global score (p=0.02) and social interaction (p=0.019) improved in treated group compared to controls	No serious adverse events
Bent et al., 2020 (Bent, Chen et al. 2020)	12 patients with ASD, Ages 13-19 yo (10 boys, 2 girls)	Prospective, open label, *d,l*-leucovorin 2 mg/kg/day (max 50 mg/day) for 12 weeks	ABC: 2.4-point improvement (p=0.56) SRS: 7.8-point improvement (p=0.095) PedsQL: 0.8-point worsening (p=0.69)	Limitations included heterogenous population and small sample size. Generally, well tolerated.
Adams et al., 2021 (Adams, Bhargava et al. 2021)	1,286 participants with ASD or their parents/caregivers	Survey of treatments including *dl*-leucovorin	Folinic acid (more than 5 mg/day orally) improved cognition in 33%, attention in 29%, and language/communication in 24%. Moderate dose folinic acid (below 5 mg/day orally) improved language/communication (20%)	Minimal adverse effects reported

Autism Diagnostic Observation Schedule (ADOS)

Table S7. Studies examining *d,l*-leucovorin along with other supplements or treatments in ASD, by year published

Study, year	Children	Study and dose	Outcomes	Comment
James et al., 2004 (James, Cutler et al. 2004)	8 children with ASD	Prospective, open label, 800 µg of *d,l*-leucovorin plus 1000 mg of betaine twice a day for 4 months	Increases in methionine, SAM, homocysteine, cystathionine, cysteine, and tGSH concentrations and SAM:SAH and tGSH:GSSG; improvements in speech and cognition	Clinical improvements noted by attending physician by not formally quantified
James et al., 2009 (James, Melnyk et al. 2009)	40 children with ASD	Prospective, open label, 400 µg *d,l*-leucovorin twice a day and 75 µg/kg methylcobalamin injected twice a week	Significant increases in cysteine, cysteinylglycine, and glutathione concentrations ($p<0.001$)	Side effects included hyperactivity and reduced sleep
Adams, 2011 (Adams, Audhya et al. 2011)	141 children with ASD; 72 received MVI/minerals; 69 received placebo	Double-blind, placebo-controlled study; MVI containing 550 µg of *d,l*-leucovorin per day for 3 months	Improvements in PGI-R overall ($p=0.02$), hyperactivity ($p=0.003$), tantrumming ($p=0.009$), and receptive language ($p=0.03$)	MVI/minerals contained vitamins A, D, E, K, B1-B6, B12, CoQ10, and minerals
Frye et al., 2013 (Frye, Melnyk et al. 2013)	40 children with ASD; 37 analyzed	Prospective, open label, 400 µg *d,l*-leucovorin twice a day and 75 µg/kg methylcobalamin injected twice a week	Significant improvements in receptive ($p=0.001$), expressive ($p<0.0001$) and written ($p<0.005$) communication skills; personal ($p<0.0005$), domestic ($p<0.05$) and community ($p<0.005$) daily living skills; and interpersonal ($p<0.05$), play-leisure ($p=0.001$), and coping ($p=0.0005$) social skills	Additional analysis of James, 2009 (James, Melnyk et al. 2009). Side effects included hyperactivity (14%), reduced sleep (7%), insomnia (2%), impulsivity (2%) and irritability (2%)
Adams et al., 2018 (Adams, Audhya et al. 2018)	67 individuals with ASD (ages 3-58 yo); 37 received MVI/minerals; 30 were untreated	Prospective controlled study; MVI containing *d,l*-leucovorin, folic acid and 5MTHF (600 µg) per day for 12 months	Improvements in non-verbal IQ ($p=0.009$); CARS score ($p=0.03$), VABS-II communication ($p=0.01$), daily living skills ($p=0.007$), and social skills ($p=0.05$)	MVI/minerals contained vitamins A, D, E, K, B1-B6, B12, CoQ10, and minerals; 2 children had worsening behaviors

Ramaekers et al., 2019 (Ramaekers, Sequeira et al. 2019)	166 patients with ASD, ages 1-16 yo (82 treated; 84 untreated, matched for age, gender, CARS score and FRα autoantibody status)	Prospective open label, controlled study; *d,l*-leucovorin 0.5-2 mg/kg/day for 2 years	Compared to untreated control group, *d,l*-leucovorin led to improvements in CARS scores from severe ASD to mild or moderate ASD; 17/82 (20.7%) had "complete recovery"	Other nutritional deficiency corrected during the study period
Batebi (Batebi, Moghaddam et al. 2021)	28 children with ASD treated (mean age 8.36±1.81); 27 children with ASD receiving placebo (7.52±1.84)	*d,l*-leucovorin 2 mg/kg/day up to 50 mg per day added to risperidone	Improvements in ABC-C stereotypy (p=0.034), hyperactivity/noncompliance (p=0.03)	Common side effects were increase appetite (25%) and diarrhea (18%)

Parental Global Impressions-Revised (PGI-R)

References

1. Adams, J. B., T. Audhya, E. Geis, E. Gehn, V. Fimbres, E. L. Pollard, J. Mitchell, J. Ingram, R. Hellmers, D. Laake, J. S. Matthews, K. Li, J. C. Naviaux, R. K. Naviaux, R. L. Adams, D. M. Coleman and D. W. Quig (2018). "Comprehensive Nutritional and Dietary Intervention for Autism Spectrum Disorder-A Randomized, Controlled 12-Month Trial." Nutrients 10(3).
2. Adams, J. B., T. Audhya, S. McDonough-Means, R. A. Rubin, D. Quig, E. Geis, E. Gehn, M. Loresto, J. Mitchell, S. Atwood, S. Barnhouse and W. Lee (2011). "Effect of a vitamin/mineral supplement on children and adults with autism." BMC Pediatr 11: 111.
3. Adams, J. B., A. Bhargava, D. M. Coleman, R. E. Frye and D. A. Rossignol (2021). "Ratings of the Effectiveness of Nutraceuticals for Autism Spectrum Disorders: Results of a National Survey." J Pers Med 11(9): 878.
4. Al-Baradie, R. S. and M. W. Chaudhary (2014). "Diagnosis and management of cerebral folate deficiency. A form of folinic acid-responsive seizures." Neurosciences (Riyadh) 19(4): 312-316.
5. Batebi, N., H. S. Moghaddam, A. Hasanzadeh, Y. Fakour, M. R. Mohammadi and S. Akhondzadeh (2021). "Folinic Acid as Adjunctive Therapy in Treatment of Inappropriate Speech in Children with Autism: A Double-Blind and Placebo-Controlled Randomized Trial." Child Psychiatry Hum Dev 52(5): 928-938.
6. Bent, S., Y. Chen, M. G. McDonald, F. Widjaja, J. Wahlberg and R. L. Hendren (2020). "An Examination of Changes in Urinary Metabolites and Behaviors with the Use of Leucovorin Calcium in Children with Autism Spectrum Disorder (ASD)." Advances in Neurodevelopmental Disorders 4(3): 241-246.
7. Frye, R. E., L. Delhey, J. Slattery, M. Tippett, R. Wynne, S. Rose, S. G. Kahler, S. C. Bennuri, S. Melnyk, J. M. Sequeira and E. Quadros (2016). "Blocking and Binding Folate Receptor Alpha Autoantibodies Identify Novel Autism Spectrum Disorder Subgroups." Front Neurosci 10: 80.
8. Frye, R. E., S. Melnyk, G. Fuchs, T. Reid, S. Jernigan, O. Pavliv, A. Hubanks, D. W. Gaylor, L. Walters and S. J. James (2013). "Effectiveness of methylcobalamin and folinic Acid treatment on adaptive behavior in children with autistic disorder is related to glutathione redox status." Autism Res Treat 2013: 609705.
9. Frye, R. E. and R. K. Naviaux (2011). "Autistic disorder with complex IV overactivity: a new mitochondrial syndrome." Journal of Pediatric Neurology 9(4): 427-434.
10. Frye, R. E., J. M. Sequeira, E. V. Quadros, S. J. James and D. A. Rossignol (2013). "Cerebral folate receptor autoantibodies in autism spectrum disorder." Mol Psychiatry 18(3): 369-381.
11. Frye, R. E., J. M. Sequeira, E. V. Quadros and D. A. Rossignol (2014). "Folate Receptor Alpha Autoantibodies Modulate Thyroid Function in Autism Spectrum Disorder." N A J Med Sci 7(2): 53-56.
12. Frye, R. E., J. Slattery, L. Delhey, B. Furgerson, T. Strickland, M. Tippett, A. Sailey, R. Wynne, S. Rose, S. Melnyk, S. Jill James, J. M. Sequeira and E. V. Quadros (2018). "Folinic acid improves verbal communication in children with autism and language impairment: a randomized double-blind placebo-controlled trial." Mol Psychiatry 23(2): 247-256.
13. Frye, R. E., R. Wynne, S. Rose, J. Slattery, L. Delhey, M. Tippett, S. G. Kahler, S. C. Bennuri, S. Melnyk, J. M. Sequeira and E. V. Quadros (2017). "Thyroid dysfunction in children with autism spectrum disorder is associated with folate receptor alpha autoimmune disorder." J Neuroendocrinol 29(3).

14. James, S. J., P. Cutler, S. Melnyk, S. Jernigan, L. Janak, D. W. Gaylor and J. A. Neubrander (2004). "Metabolic biomarkers of increased oxidative stress and impaired methylation capacity in children with autism." Am J Clin Nutr 80(6): 1611-1617.
15. James, S. J., S. Melnyk, G. Fuchs, T. Reid, S. Jernigan, O. Pavliv, A. Hubanks and D. W. Gaylor (2009). "Efficacy of methylcobalamin and folinic acid treatment on glutathione redox status in children with autism." Am J Clin Nutr 89(1): 425-430.
16. Kanmaz, S., E. Simsek, S. Yilmaz, A. Durmaz, H. M. Serin and S. Gokben (2021). "Cerebral folate transporter deficiency: a potentially treatable neurometabolic disorder." Acta Neurol Belg.
17. Moretti, P., S. U. Peters, D. Del Gaudio, T. Sahoo, K. Hyland, T. Bottiglieri, R. J. Hopkin, E. Peach, S. H. Min, D. Goldman, B. Roa, C. A. Bacino and F. Scaglia (2008). "Brief report: autistic symptoms, developmental regression, mental retardation, epilepsy, and dyskinesias in CNS folate deficiency." J Autism Dev Disord 38(6): 1170-1177.
18. Moretti, P., T. Sahoo, K. Hyland, T. Bottiglieri, S. Peters, D. del Gaudio, B. Roa, S. Curry, H. Zhu, R. H. Finnell, J. L. Neul, V. T. Ramaekers, N. Blau, C. A. Bacino, G. Miller and F. Scaglia (2005). "Cerebral folate deficiency with developmental delay, autism, and response to folinic acid." Neurology 64(6): 1088-1090.
19. Quadros, E. V., J. M. Sequeira, W. T. Brown, C. Mevs, E. Marchi, M. Flory, E. C. Jenkins, M. T. Velinov and I. L. Cohen (2018). "Folate receptor autoantibodies are prevalent in children diagnosed with autism spectrum disorder, their normal siblings and parents." Autism Res 11(5): 707-712.
20. Ramaekers, V. T. and N. Blau (2004). "Cerebral folate deficiency." Dev Med Child Neurol 46(12): 843-851.
21. Ramaekers, V. T., N. Blau, J. M. Sequeira, M. C. Nassogne and E. V. Quadros (2007). "Folate receptor autoimmunity and cerebral folate deficiency in low-functioning autism with neurological deficits." Neuropediatrics 38(6): 276-281.
22. Ramaekers, V. T., E. V. Quadros and J. M. Sequeira (2013). "Role of folate receptor autoantibodies in infantile autism." Mol Psychiatry 18(3): 270-271.
23. Ramaekers, V. T., S. P. Rothenberg, J. M. Sequeira, T. Opladen, N. Blau, E. V. Quadros and J. Selhub (2005). "Autoantibodies to folate receptors in the cerebral folate deficiency syndrome." N Engl J Med 352(19): 1985-1991.
24. Ramaekers, V. T., J. M. Sequeira, N. Blau and E. V. Quadros (2008). "A milk-free diet downregulates folate receptor autoimmunity in cerebral folate deficiency syndrome." Dev Med Child Neurol 50(5): 346-352.
25. Ramaekers, V. T., J. M. Sequeira, M. DiDuca, G. Vrancken, A. Thomas, C. Philippe, M. Peters, A. Jadot and E. V. Quadros (2019). "Improving Outcome in Infantile Autism with Folate Receptor Autoimmunity and Nutritional Derangements: A Self-Controlled Trial." Autism Res Treat 2019: 7486431.
26. Ramaekers, V. T., J. M. Sequeira, B. Thony and E. V. Quadros (2020). "Oxidative Stress, Folate Receptor Autoimmunity, and CSF Findings in Severe Infantile Autism." Autism Res Treat 2020: 9095284.
27. Renard, E., B. Leheup, R. M. Gueant-Rodriguez, A. Oussalah, E. V. Quadros and J. L. Gueant (2020). "Folinic acid improves the score of Autism in the EFFET placebo-controlled randomized trial." Biochimie 173: 57-61.
28. Shoffner, J., L. Hyams, G. N. Langley, S. Cossette, L. Mylacraine, J. Dale, L. Ollis, S. Kuoch, K. Bennett, A. Aliberti and K. Hyland (2010). "Fever plus mitochondrial disease could be risk factors for autistic regression." J Child Neurol 25(4): 429-434.
29. Shoffner, J., B. Trommer, A. Thurm, C. Farmer, W. A. Langley, 3rd, L. Soskey, A. N. Rodriguez, P. D'Souza, S. J. Spence, K. Hyland and S. E. Swedo (2016). "CSF concentrations of 5-methyltetrahydrofolate in a cohort of young children with autism." Neurology 86(24): 2258-2263.
